# Bats of Georgia - an occurrence dataset from 1835 through 2022

**DOI:** 10.3897/BDJ.11.e103181

**Published:** 2023-06-09

**Authors:** Ioseb Natradze, Alexander Buknikashvili, Giorgi Sheklashvili, Levan Mumladze

**Affiliations:** 1 Institute of Zoology of Ilia State University, Tbilisi, Georgia Institute of Zoology of Ilia State University Tbilisi Georgia

**Keywords:** Bats, Chiroptera, dataset, distribution, Caucasus, Georgia

## Abstract

**Background:**

In Georgia, currently, 30 species of bats are recorded from four families and eleven genera. Although the oldest record of bats is from 1835 and continues until today, there are no comprehensive data available for bat diversity and distribution in Georgia. Thus, we aimed to fill that gap and make complete, expertly curated literature and our own published data openly available (through GBIF) for researchers and conservationists.

**New information:**

In this publication, out of 1987 records, 1243 (62.4%) are new and unpublished data. Generally, out of all records, 34% are literature and museum data and 66% are data collected by us. Additionally, for the first time in the history of the study of bats in Georgia, we initiated surveys in forested areas of the country.

## Introduction

Georgia is a part of the Caucasus biodiversity hotspot - one of 36 biodiversity hotspots recognised in the world, with incredible landscape and species diversity (Myers et al. 2000; Mittermeier et al. 2011; Noss et al. 2014). Although Georgia, as a part of the Caucasus, was distinguished as a biodiversity hotspot 22 years ago, the diversity and distribution of animal species remains poorly investigated (Mumladze et al. 2020). Even the inventory of vertebrate taxa (that were used by Myers et al. (2000) along with plants to delineate biodiversity hotspots) is not yet satisfactorily done, leaving room to improve the understanding of species diversity, distribution and ecological requirements.

The oldest documented record of bats in Georgia dates back to 1835 when Nordmann (1840) reported four species. This was followed by surveys conducted by Kolenati in 1843 (Kolenati 1860). After that and for nearly 40 years, no new data were collected until the beginning of the 20^th^ century (Radde 1899; Satunin 1903; Satunin 1908; Satunin 1912; Satunin 1915). As a result, 16 species for Georgia were indicated. Bat research even in the 20^th^ century was not active, rather was sporadic and information was scattered in different Georgian and Russian publications: Chkhikvishvili (1926); Ognev 1928; Kuzyakin (1950); Janashvili (1953); Janashvili (1963); and these publications report 22 species for Georgia. Sporadic information about bat species occurrence is also given in the publications by non-bat researchers including Papava 1949; Papava 1953; Papava 1960; Matsaberidze 1961; Avaliani (1963); Matsaberidze and Khotenovsky 1966; Matsaberidze and Khotenovskii 1967; Avaliani (1969a); Avaliani (1969b); Avaliani (1970); Avaliani (1973); Perov 1975; Avaliani (1976); Matsaberidze (1976); Perov 1980; Perov (1983); Strelkov (1983); Strelkov (1988); and Shidlovsky (2013).

Systematic surveys of the bat fauna of Georgia were started at the end of the 20^th^ century and resulted in a number of publications (Bukhnikashvili and Kandaurov 2002; Ivanitsky 2002; Bukhnikashvili et al. 2004; Gazaryan and Bukhnikashvili 2005; Bukhnikashvili et al. 2008; Gazaryan et al. 2008; Yavruyan et al. 2008; Bukhnikashvili et al. 2009; Ivanitsky 2010; Gazaryan et al. 2017; Ivanitsky 2017; Ivanitsky 2018; Imnadze et al. 2020; Urushadze et al. 2021). As a result of these studies, the number of bat species recorded in Georgia increased to 26 (Bukhnikashvili 2004). However, a considerable part of the results of bat surveys conducted during the last 25 years are either published in grey literature (i.e. publicly unavailable project reports) or kept unpublished by the authors. In addition to distribution data for other species, our unpublished data also provides records of four additional species (*Rhinolophusblasii*, *Myotisalcathoe*, *Myotisdavidii* and *Tadaridateniotis*) in Georgia that have not been previously documented in the literature. Thus, the goal of the present publication was to consolidate all available data about bat records of Georgia from 1835 through to 2022 into a comprehensive dataset and make it available through global and open-source databases such as GBIF (GBIF.org 2023) in order to facilitate further research and conservation of bats in Georgia.

## Sampling methods

### Study extent

**Figure 1.**
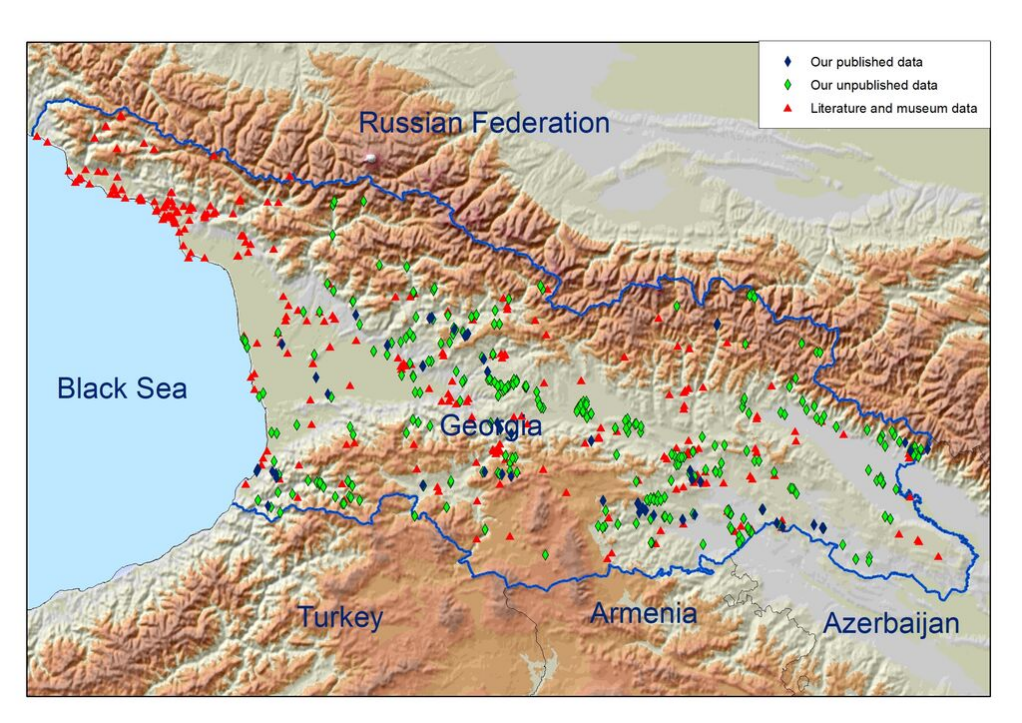
Bat records in Georgia from 1835 through to 2023.

The dataset, prepared by Natradze et al. (2023), contains information about 1987 records of 30 species of four families and 11 genera collected from 1835 through to 2023 in country of Georgia (Fig. 1).

### Sampling description

These records are based on literature published in Georgian, Russian and English languages, as well as data collected in the field by the authors during the last three decades. To collect field data, we employed various methods, including mist-netting, harp traps, visual inspection of both artificial and natural underground and overground habitats and other shelters (Dietz and Kiefer 2016). Additionally, we used hand-held bat detectors, specifically the Pettesson D240x ultrasound bat model. In our dataset, we use data obtained through hand-held bat detectors, which were validated by visually confirming the presence of the bat. To ensure high data quality, all recorded data were included only if bats were identified at the species level.

### Quality control

In the dataset, 37.4% of records are based on literature. For each of the literature records, we retrieved as much information as possible, such as sampling date, location, closest populated area, habitat etc. For most of the literature data (especially old ones), no exact geographic coordinates were given. However, since the vernacular names of sampling areas (i.e. names of subterranean objects) along with habitat descriptions were provided in many cases, we were able to exactly georeference a large number of sampling locations for 55.9% of literature records. On the other hand, not all records in literature are supplied with sampling dates and we were able to retrieve information on sampling dates for only 81.6% of literature records.

In the dataset, we provide location common names for all records. Geographic coordinates with 4 m accuracy are given for most of the records (74%), while for 26% (all literature data) of records, we have coordinates without accurate information. Additionally, 9% of records are given without the collecting dates. Record summary by species is given in Table 1, while the species records with metadata are provided at the GBIF web portal (Natradze et al. 2023).

Our database contains several cases that require further clarification regarding the identity of certain species, including: (i) some records of *Myotisnattereri* may actually pertain to *M.tschuliensis*, as suggested by Çoraman et al. (2019) and Kruskop and Solovyeva (2020); (ii) the identification of *M.davidii*/*mystacinus* may be erroneous due to their cryptic nature; and (iii) the potential existence of another species, *Miniopteruspallidus*, in eastern Georgia, as proposed by Šrámek et al. (2012). However, additional research, including DNA analysis, is required to confirm the identity of these species. Any modifications resulting from these investigations will be reflected in the subsequent version of the dataset.

## Geographic coverage

### Description

The presented bat distribution dataset originated from the whole Georgian territory. Georgia (Fig. 1), covering an area of 69,700 km^2^, is located on the southern slopes of the Great Caucasus Mountain Range, Lesser Caucasus Mountains on the isthmus between the Black and Caspian Seas. It contains lowlands between the above-mentioned mountain ranges which include the Colchis lowland in the west (along the Black Sea Coast) and the Kura River lowland in the east. The land of Georgia covers an elevation range from sea level to approximately 5,184 m at Mount Shkhara. Two-thirds of the country is mountainous with an average height of 1200 m a.s.l.

Due to its diverse geography, the climate of the region varies greatly, from very humid lowlands and mountain forests in the west to dry forests and semi-deserts in the east and glaciated nival belts in the north. There are two zoogeographic subzones and three zoogeographic provinces in Georgia: the Circumboreal subzone (the Caucasus district of the European forest province) and Mediterranean subzone (the Anterior Asia district of the Mediterranean province and the Kura district of the Iran-Turan province) (Verestchagin 1959). Georgia has about 72 types of landscapes (Beruchashvili 2000): humid sub-tropic landscapes are in the western part; alpine landscapes are spread in the northern and north-eastern part; the typical Middle East treeless uplands are presented in the southern part; and semi-deserts of the Turanian type in the southeast part of the country.

The studied territory contains diverse bat habitats. Along with various kinds of forests (temperate broadleaf, evergreen and dry forests), particularly relevant are the western Great Caucasus slopes, which are represented by a number of Karst massifs that provide a large and diverse (yet only partly explored) subterranean environment suitable for bat species.

### Coordinates

40.946 and 43.818 Latitude; 39.660 and 46.933 Longitude.

## Temporal coverage

### Notes

The dataset includes data collected from 1835 through to 2023. For all other records, time coverage could be divided into the following time periods, data collected in (i) 19^th^, (ii) 20^th^ and (iii) 21^st^ centuries. In the 19^th^ century, there are 53 records which make up 2.7% of all records; in the 20^th^ century, there are 231 records which make up 11.6% of all records and in the 21^st^ century, there are 1523 records, which make up 76.7%. From the 1523 records, made in the 21^st^ century, 1239 records are new, unpublished records, which make up 62.4% of all records.

## Usage licence

### Usage licence

Open Data Commons Attribution License

## Data resources

### Data package title

Bats of Georgia

### Resource link

https://www.gbif.org/dataset/8e1c23ba-5618-4bba-8fbf-a195cce8dda0

### Number of data sets

1

### Data set 1.

#### Data set name

batsofgeorgia

#### Data format

Excel table

#### Data format version

V1.2

**Data set 1.** 

Column label	Column description
occurrenceID	Unique identifier of record.
kingdom	The full scientific name of the kingdom in which the taxon is classified.
phylum	The full scientific name of the phylum in which the taxon is classified.
class	The full scientific name of the class in which the taxon is classified.
order	The full scientific name of the order in which the taxon is classified.
family	The full scientific name of the subfamily in which the taxon is classified.
scientificName	Species full scientific (Latin) name including authorship and year.
locality	The specific description of the place of collection.
eventDate	Collection event date.
countryCode	Standard ISO 3166-1-alpha-2 country code
decimalLatitude	The geographic latitude (in decimal degrees).
decimalLongitude	The geographic longitude (in decimal degrees).
geodeticDatum	Geographic coordinates reference system EPSG.
coordinateUncertaintyInMetres	Coordinate measurement accuracy (metres in case of GPS recordings, NA - if manually georeferenced). However, see the field "dataGeneralisations" for furher details
minimumElevationInMetres	Minimum elevation above sea level.
maximumElevationInMetres	Maximum elevation above sea level.
associatedReferences	Source for the particular record.
georeferenceSources	The system used during the georeferencing.
dataGeneralisations	According to the "Agreement on the Conservation of Populations of European Bats" (Eurobats, United Nations Environment Programme, Eurobats, May 2019), (Retrieved 7 August 2019), we intentionally reduced the precision of geographic coordinates in the dataset, while more precise information is available upon request.
basisOfRecord	The specific nature of the data record.
institutionCode	The code of the institution where data are stored.
collectionCode	The code of the collection.

## Additional information

Field data were collected under the permissions #2722/01; 2302/01; R/057-21, issued by the Ministry of Environmental Protection and Agriculture of Georgia.

## Figures and Tables

**Table 1.** Number of records for each species.

#	Species	Total Number of records	Literature & Museum data	Our published & unpublished data	Unpublished data
	**Chiroptera**				
	**Rhinolophidae**				
	**Rhinolophus**				
1	*Rhinolophusferrumequinum* (Schreber, 1774)	185	58	127	84
2	*Rhinolophushipposideros* (André, 1797)	167	83	84	53
3	*Rhinolophuseuryale* Blasius, 1853	51	20	31	23
4	*Rhinolophusblasii* Peters, 1866	6	0	6	5
5	*Rhinolophusmehelyi* Matschie, 1901	5	5	0	0
	**Vespertilionidae**				
	**Myotis**				
6	*Myotisblythii* (Tomes, 1857)	120	34	86	61
7	*Myotisbechsteinii* Kuhl, 1817	25	4	21	21
8	Myotisdaubentonii (Kuhl, 1817)	41	6	35	33
9	*Myotisnatereri* (Kuhl, 1817)	65	12	53	51
10	*Myotisemarginatus* (Geoffroy, 1806)	72	15	57	35
11	*Myotisalcathoe* von Helversen & Heller, 2001	25	1	24	24
12	*Myotisbrandtii* (Eversmann, 1845)	28	3	25	24
13	*Myotisdavidii* (Peters, 1869)	3	0	3	3
14	*Myotismystacinus* (Kuhl, 1817)	89	33	56	54
	**Nyctalus**				
15	*Nyctalusnoctula* (Schreber, 1774)	89	16	73	72
16	*Nyctalusleisleri* (Kuhl, 1817)	87	10	77	68
17	*Nyctaluslasiopterus* (Schreber, 1780)	23	7	16	8
	**Eptesicus**				
18	*Eptesicusnilssonii* (von Keyserling & Blasius, 1839)	4	1	3	2
19	*Eptesicusserotinus* (Schreber, 1774)	160	48	112	105
	**Pipistrellus**				
20	*Pipistrelluspipistrellus* (Schreber, 1774)	289	64	225	218
21	*Pipistrelluspygmaeus* (Leach, 1825)	74	0	74	70
22	*Pipistrelluskuhlii* (Kuhl, 1817)	83	11	72	70
23	*Pipistrellusnathusii* (von Keyserling & Blasius, 1839)	17	15	2	2
	**Hypsugo**				
24	*Hypsugosavii* (Bonaparte, 1837)	23	4	19	17
	**Barbastella**				
25	*Barbastellabarbastellus* (Schreber, 1774)	63	8	55	49
	**Plecotus**				
26	*Plecotusauritus* (Linnaeus, 1758)	60	29	31	29
27	*Plecotusmacrobularis* Kuzyakin, 1965	14	5	9	8
	**Vespertilio**				
28	*Vespertiliomurinus* Linnaeus, 1758	38	15	23	23
	**Miniopteridae**				
	**Miniopterus**				
29	*Miniopterusschreibersii* (Kuhl, 1817)	65	35	30	18
	**Molossidae**				
	**Tadarida**				
30	*Tadaridateniotis* (Rafinesque, 1814)	14	1	13	13
